# Can health justice be measured? A normative reconstruction of health equity indicators

**DOI:** 10.1186/s13010-026-00234-1

**Published:** 2026-09-23

**Authors:** Akbar Shahrivari

**Affiliations:** School of Traditional Medicine, Shahid Beheshti University, Tehran, Islamic Republic of Iran

**Keywords:** Health justice, Health equity, Health equity indicators, Theories of justice, Normative evaluation, Justificatory attribution, Structural justice, Capability approach

## Abstract

**Background:**

Health systems commonly evaluate justice using quantitative health equity indicators such as healthcare coverage, financial protection, population health outcomes, and measures of the social determinants of health. These indicators are often treated as competing operationalisations of a single moral standard. This article argues that this assumption is conceptually mistaken because different indicators evaluate different normative objects rather than alternative dimensions of the same conception of justice.

**Methods:**

Using a comparative normative methodology, the article analyses six theories of justice that have significantly shaped contemporary debates in biomedical ethics and health policy. Through the principles of justificatory attribution and the Focal Attribution Procedure, families of health equity indicators are systematically attributed to the normative domains that provide their primary moral justification.

**Results:**

The analysis suggests that widely used health equity indicators do not approximate a single moral construct. Instead, they identify distinct evaluative objects corresponding to different conceptions of justice, including aggregate health welfare, fair opportunity, responsibility-sensitive disadvantage, collective health goods, human functioning, and structural disadvantage. Persistent disagreement over health equity indicators therefore reflects normative pluralism rather than empirical or methodological inadequacy. Building on this analysis, the article proposes a Layered Evaluation Model that preserves the conceptual distinction between normative domains while providing a coherent framework for interpreting health equity indicators.

**Conclusion:**

Because the normative domains of health justice are conceptually distinct, representing health justice through a single composite index risks obscuring the evaluative objects that different theories seek to protect unless the weights used to construct that index are made explicit and normatively defended. Health equity measurement should therefore be understood as a process of explicit normative interpretation rather than technical aggregation. By systematically relating empirical indicators to their primary justificatory foundations, the proposed framework offers a more conceptually coherent, philosophically grounded, and policy-relevant approach to the evaluation of health justice.

## Introduction

Disagreement over the measurement of health justice has persisted despite substantial advances in health information systems and health equity metrics. New indicators have been developed, existing measures have been refined, and increasingly sophisticated frameworks have been proposed for evaluating health systems. Nevertheless, fundamental controversies remain unresolved. Debate continues over whether health justice is best evaluated through population health outcomes, healthcare access, financial protection, individual responsibility, human functioning, or the social determinants of health. The persistence of these disagreements suggests that the central challenge is not simply methodological but conceptual. It raises a prior philosophical question concerning the normative object that health equity indicators are intended to measure [1–3].

Most approaches to health equity measurement implicitly assume that competing indicators represent alternative operationalisations of a single concept of justice. Under this assumption, disagreement is interpreted primarily as a technical problem, and differences between indicators are understood as reflecting alternative methods of measuring the same underlying moral construct. Consequently, methodological efforts have focused on improving measurement techniques, refining indicator sets, and increasing the validity and policy relevance of empirical assessment. Although these developments have substantially strengthened the measurement of health inequalities, they have not resolved the underlying normative disagreement concerning what health justice itself requires [1, 4, 5].

This article argues that the prevailing assumption is mistaken. Health equity indicators do not compete because they measure the same normative object differently. Rather, they evaluate different normative objects defined by different theories of justice. Population health, healthcare access, responsibility-sensitive disadvantage, collective health goods, human functioning, and structural disadvantage do not merely represent different dimensions of a single conception of justice. Instead, each identifies a distinct moral reason for regarding a health inequality as unjust. Persistent disagreement over health equity indicators therefore reflects normative pluralism rather than methodological inadequacy [6–10].

The aim of this article is neither to defend one theory of justice against competing alternatives nor to develop another composite measure of health equity. Instead, it reconstructs the principal normative domains underlying contemporary theories of justice in health and examines how each provides the primary justificatory basis for a distinct family of health equity indicators. To achieve this objective, the article develops the principle of justificatory attribution, according to which indicator families are assigned to the normative domain whose theory provides the primary moral justification for interpreting the inequality they reveal as unjust. Building upon this principle, the article introduces a Focal Attribution Procedure for the analytical attribution of indicator families and proposes a Layered Evaluation Model that preserves normative plurality while avoiding the conceptual reduction inherent in composite measures [7, 9, 10].

The remainder of the article is organised as follows. The next section presents the methodological framework, explains why health equity indicators require normative interpretation, and justifies the selection of the theories included in the analysis. The article then reconstructs the principal normative domains of health justice and examines how each domain provides the justificatory basis for a distinct family of health equity indicators. Finally, it develops a layered model of health justice evaluation and discusses its implications for health equity measurement, policy analysis, and priority-setting.

## Methodological framework

### Why health equity indicators require normative interpretation

Health equity indicators are commonly presented as empirical measures of justice. Yet, considered independently, indicators describe patterns of health, healthcare, or social conditions without determining why those patterns should be regarded as matters of justice. Mortality rates, healthcare utilisation, financial hardship, functional limitations, or social deprivation become indicators of health justice only when interpreted within a normative framework that specifies which inequalities are morally significant and the reasons for their moral significance [2, 3, 7].

Accordingly, disagreement over health equity indicators cannot be understood solely as disagreement about measurement. It also reflects disagreement concerning the normative interpretation of health inequalities. Before indicators can evaluate justice, they must first be interpreted through a conception of justice. The methodological framework developed in this article is designed to make this interpretive step explicit [5, 7, 9].

### Methodological orientation

This study adopts a comparative normative methodology, understood specifically as a rational reconstruction: an interpretive exercise that reconstructs the internal logic of established theories of justice as applied to health, rather than an empirical study, a purely exegetical history of ideas, or an attempt to derive a new theory through reflective equilibrium. It does not seek to evaluate the empirical validity of existing health equity indicators, nor does it attempt to determine which theory of justice is philosophically superior. Instead, its objective is to reconstruct the distinct evaluative objects identified by contemporary theories of justice and to explain how these evaluative objects provide the primary normative justification for different families of health equity indicators. The analysis therefore focuses on normative justification rather than empirical measurement [7, 9, 10].

The adequacy of this reconstruction should be assessed against three standards: fidelity to the canonical texts from which each theory is drawn, internal consistency in the application of the justificatory attribution principle across domains, and coverage of the indicator families most frequently used in contemporary health-policy practice. The analysis does not claim completeness against every possible theory of justice or every indicator in use; it claims that, for the theories and indicators it does address, the attribution offered is defensible on these three grounds.

### Selection of theories

The analysis is limited to the principal theories of justice that have significantly influenced contemporary debates in biomedical ethics and health policy. This prominence is evidenced by their recurring use as organising frameworks in major syntheses of health justice, including Beauchamp and Childress’s principlism, Daniels’s application of Rawlsian theory to health, Powers and Faden’s structural account, and Ruger’s health capability paradigm, each of which surveys multiple competing theories of justice as applied to health [7, 9–11]. Luck egalitarianism and communitarianism are included because they are the two traditions most frequently invoked, respectively, to justify responsibility-sensitive health policy and collective public-health measures in the applied bioethics literature [12–14]. The selection is therefore purposive rather than exhaustive: it draws on theories with an established presence in the health-policy literature rather than a comprehensive taxonomy of political philosophy, and it does not claim that other theories, prioritarianism, sufficientarianism, or feminist ethics of care, for example, are without relevance to health justice.

Rather than proposing a comprehensive taxonomy of political theories of justice, the article builds upon the principal theories of justice discussed in contemporary biomedical ethics, taking the framework presented by Beauchamp and Childress as its point of departure rather than as a taxonomy to be reproduced. For the analytical purposes of this study, the egalitarian tradition is reconstructed into two distinct normative domains, liberal egalitarianism and luck egalitarianism, because they identify different evaluative objects and justify different families of health equity indicators: liberal egalitarianism evaluates inequalities primarily in terms of fair opportunity, whereas luck egalitarianism evaluates them according to the distinction between brute luck and individual responsibility [6, 7, 12, 15–17]. Their analytical separation therefore serves conceptual clarification rather than taxonomic revision. Correspondingly, libertarianism is not treated as an independent domain because, unlike the other theories, it does not generate a distinctive family of health equity indicators within the justificatory attribution framework developed in this study [10].

The family of indicators associated with each domain was compiled through a purposive narrative review of the international health-monitoring and health-economics literature associated with each theory’s evaluative object, rather than a systematic review. For the utilitarian domain, this drew on established health-economics texts and methodological standards for aggregate health measurement [18–21]. For the opportunity and structural domains, this drew on international monitoring frameworks published by the World Health Organization and the Commission on Social Determinants of Health [4, 22, 23]. An indicator was included where it is in active use in health-policy practice or international monitoring, and where its normative significance could be clearly and defensibly traced to a single domain’s evaluative object under the justificatory attribution principle set out in Section "Justificatory attribution".

### Justificatory attribution

The central methodological principle of this study is justificatory attribution. Health equity indicators are attributed according to the primary normative justification that explains why the inequality they reveal should be regarded as unjust. The analysis therefore addresses a justificatory rather than a descriptive question: What is the primary moral reason that makes the inequality identified by a given indicator an injustice rather than merely an unfortunate difference?

The same empirical indicator may legitimately be interpreted within more than one theory of justice. Mortality, for example, may be regarded as morally significant because it reduces aggregate welfare, restricts fair opportunity, limits human functioning, or reflects structural disadvantage. The purpose of justificatory attribution is not to deny these alternative interpretations but to identify the normative framework that provides the primary moral justification for interpreting the inequality revealed by a particular indicator as an injustice [7–9].

A second example illustrates the same procedure in a domain where the primary attribution is less immediately obvious. Vaccination coverage may be interpreted as significant under several theories: a utilitarian may value it because widespread immunisation reduces aggregate disease burden; a liberal egalitarian may value it because unequal access restricts fair opportunity for the unvaccinated; and a communitarian may value it because immunity is a good that can only be secured collectively, since an individual’s protection depends on the vaccination status of others through herd immunity. Under the Focal Attribution Procedure, vaccination coverage is attributed to the communitarian domain because its primary moral significance depends on a feature the other two interpretations do not capture: the good it protects is not reducible to the sum of individual protections and cannot be secured by any single person’s choice, however resourced. The utilitarian and liberal-egalitarian readings remain available as secondary interpretations, particularly where coverage gaps are patterned by socioeconomic status, but the primary justification for treating uneven vaccination coverage as unjust, rather than merely suboptimal, is the failure of a good that exists only in collective form [24, 25].

Accordingly, indicators are attributed according to justificatory priority rather than historical origin, empirical application, or policy use. The proposed framework is therefore interpretive rather than descriptive. It relates empirical indicators to the evaluative objects that provide their principal normative significance [9, 10].

### Focal attribution procedure

Because different theories of justice frequently provide overlapping interpretations of the same empirical inequalities, the analysis employs a Focal Attribution Procedure to identify the primary normative domain of each family of health equity indicators.

Under this procedure, indicators are attributed to the normative domain that provides their primary justificatory basis. An indicator family is not reassigned to a subsequent domain unless another theory provides a conceptually distinct and more fundamental normative justification for interpreting the same inequality as unjust [7, 9, 12].

The purpose of the procedure is analytical clarification rather than theoretical exclusion. It neither assumes that theories of justice form a historical sequence or normative hierarchy nor implies that they are mutually exclusive. Likewise, it does not deny that many indicators may legitimately be interpreted within multiple normative frameworks. Instead, it identifies the domain in which the primary moral significance of an indicator family is most clearly expressed [8, 10]. The outcome is a domain-based interpretive framework in which different families of health equity indicators remain conceptually distinct while collectively contributing to a layered evaluation of health justice [7, 9]. 

### Scopes and limitations

The reconstruction offered in this article rests on interpretive judgement rather than algorithmic derivation. Assigning a given family of indicators to its primary normative domain requires deciding which of several plausible readings of a theory best captures its application to health, and reasonable scholars familiar with the same primary texts could draw the boundaries differently in particular cases. The canonical readings relied upon here — for example, Rawls’s account of fair equality of opportunity or Dworkin’s distinction between brute and option luck — are themselves the subject of ongoing philosophical debate; this article adopts widely shared interpretations rather than defending a single authoritative reading against all rivals.

The selection of theories and indicators is, as noted in Section "Selection of theories", purposive rather than exhaustive. Other theories of justice, and other indicators in current policy use, could in principle be incorporated into the layered evaluation model using the same justificatory attribution principle; their omission here reflects the scope of this article rather than a judgement that they are irrelevant to health justice.

Finally, the framework developed in this article is a normative reconstruction, not an empirical study. It has not been tested through stakeholder deliberation, expert consensus exercises, or empirical validation against how policymakers or affected populations actually reason about health inequalities. Whether the six domains identified here track the concerns that matter most to those directly affected by health policy decisions is an empirical question this article does not address, and one that would usefully complement the conceptual argument offered here.

## Normative domains of health justice

The methodological framework developed in the previous section establishes that health equity indicators require normative interpretation before they can function as measures of justice. The central question is therefore not simply which indicators should be used, but which conception of justice provides the primary moral justification for interpreting a particular health inequality as unjust. Different theories answer this question by identifying different evaluative objects. The following sections reconstruct the principal normative domains of health justice and examine how each provides the primary justificatory basis for a distinct family of health equity indicators [7, 9, 10].

### Population outcomes (utilitarian justification)

Within the present framework, the relevant normative question is whether a health inequality is unjust because it reduces overall population health. Utilitarian theories answer this question by evaluating justice in terms of aggregate well-being and by requiring institutions to produce the greatest overall health benefit achievable with available resources. In the context of health policy, the relevant good is typically understood as population health itself. Accordingly, justice is assessed primarily by the extent to which health systems maximise aggregate health rather than by the distribution of health benefits among individuals [10, 26–28].

This normative orientation underlies the development of summary measures such as quality-adjusted life years (QALYs) and disability-adjusted life years (DALYs), which aggregate diverse health states into comparable units for policy evaluation. These measures enable decision-makers to compare interventions according to the amount of health they generate relative to available resources. Within this framework, allocation is regarded as just when it produces the greatest improvement in overall population health [18, 19, 21].

The primary evaluative object of this domain is aggregate health welfare. Equality of treatment is not the central moral concern. Providing treatment to one patient rather than another may therefore be justified whenever doing so produces greater aggregate health benefit. Consequently, resource-intensive interventions benefiting relatively small populations may receive lower priority than interventions generating more modest benefits for substantially larger populations [19, 27].

The corresponding family of health equity indicators includes life expectancy, healthy life expectancy, burden of disease, preventable mortality, QALYs, DALYs, and cost-effectiveness measures. Although these indicators may also be interpreted within other theories of justice, their primary justificatory attribution within the present framework derives from the fact that they evaluate aggregate health production rather than fair opportunity, responsibility-sensitive disadvantage, collective health goods, human functioning, or structural disadvantage [19, 20, 29].

The utilitarian domain therefore captures one important dimension of health justice: the efficient production of population health. It does not, however, determine whether health benefits are distributed fairly, whether inequalities reflect differences in responsibility, whether collective health goods are adequately protected, or whether structural disadvantages persist. For this reason, utilitarian indicators constitute one normative domain within the layered evaluation model rather than a comprehensive measure of health justice [9, 10].

### Opportunity (liberal egalitarian justification)

Within the present framework, the relevant normative question is whether a health inequality is unjust because it restricts individuals’ fair opportunity to pursue their life plans. Liberal egalitarian theories answer this question by maintaining that justice requires institutions to secure fair opportunities for all members of society rather than merely maximise aggregate welfare. Health is therefore morally significant because it directly influences individuals’ ability to participate in social, economic, and political life [6, 7, 30].

This understanding is developed most prominently within the liberal egalitarian tradition, particularly through Rawls’s conception of fair equality of opportunity and Daniels’s extension of this principle to health. From this perspective, disease and unequal access to healthcare are morally significant because they impair individuals’ normal opportunities. Justice therefore requires institutions to ensure equitable access to the health services necessary to preserve or restore fair opportunity [6, 7, 30].

The primary evaluative object of this domain is fair opportunity. The corresponding family of health equity indicators therefore includes measures of healthcare access, universal health coverage, service availability, unmet healthcare needs, financial protection, and other indicators that evaluate whether individuals enjoy equitable opportunities to obtain necessary healthcare [7, 22].

From the perspective of justificatory attribution, these indicators belong to the opportunity domain because their primary moral significance lies in identifying inequalities that restrict fair opportunity. Although these indicators may also be interpreted within other normative frameworks, their primary justificatory attribution derives from the liberal egalitarian principle that justice requires equitable opportunity to achieve and maintain health [6, 7].

The opportunity domain therefore identifies a dimension of health justice that cannot be captured by measures of aggregate population health alone. A health system may maximise overall health while continuing to deny fair opportunities to particular individuals or groups. For this reason, opportunity constitutes a distinct normative domain within the layered evaluation model [7, 10].

### Responsibility (luck egalitarian justification)

Within the present framework, the relevant normative question is whether a health inequality is unjust because it results from circumstances beyond an individual’s control rather than from voluntary choice. Unlike liberal egalitarianism, which evaluates inequalities primarily in terms of fair opportunity, luck egalitarianism evaluates whether individuals should bear the consequences of disadvantages for which they are, or are not, morally responsible [12, 15–17, 31].

Luck egalitarian theories distinguish between inequalities arising from brute luck, such as congenital disease, genetic predisposition, or unavoidable environmental exposure, and those associated with informed and voluntary choice. From this perspective, justice requires institutions to compensate for disadvantages resulting from brute luck while permitting responsibility to influence distributive decisions where inequalities arise from genuinely voluntary actions [12, 15–17].

The primary evaluative object of this domain is responsibility-sensitive disadvantage. Accordingly, the corresponding family of health equity indicators includes measures distinguishing unavoidable health disadvantage from inequalities associated with behavioural risk, preventable disease, or other circumstances in which questions of individual responsibility become morally relevant [12, 32].

From the perspective of justificatory attribution, these indicators belong to the responsibility domain because their primary moral significance lies in evaluating whether health inequalities warrant compensation irrespective of individual responsibility or whether responsibility may legitimately influence distributive decisions. Their primary justificatory attribution therefore depends upon the distinction between brute luck and voluntary choice rather than upon healthcare access, aggregate population health, collective health goods, human functioning, or structural disadvantage [12, 15].

The responsibility domain therefore identifies a dimension of health justice that cannot be reduced either to aggregate welfare or to fair opportunity. Individuals may possess similar opportunities for health while differing substantially in the extent to which their health conditions result from brute luck or voluntary choice. For this reason, responsibility constitutes a distinct normative domain within the layered evaluation model [10, 12].

### Collective health goods (communitarian justification)

Within the present framework, the relevant normative question is whether a health inequality is unjust because it undermines health goods that can only be achieved and sustained collectively. Unlike the preceding domains, which primarily evaluate individual welfare, opportunity, or responsibility, this domain concerns health goods whose value depends upon collective action, shared institutions, and social cooperation [10, 25, 33].

This is not to say that communitarianism provides the only possible justification for such measures: utilitarian reasoning can support vaccination programmes on aggregate-welfare grounds, and liberal or rights-based reasoning can support them as protections of others’ opportunity or bodily security. The communitarian framing is adopted here because it identifies a feature of these goods that the other theories do not foreground as their primary concern, that the good in question is irreducibly collective, so that no individual’s protection can be secured by that individual’s own action alone [25, 33, 34].

The primary evaluative object of this domain is collective health goods. These include forms of protection whose benefits are inherently shared, such as vaccination programmes, communicable disease control, environmental sanitation, food safety, and other public health measures that cannot be secured through individual action alone [10, 24].

The corresponding family of health equity indicators therefore includes vaccination coverage, communicable disease surveillance, environmental health indicators, sanitation measures, public health preparedness, and other indicators that evaluate the effectiveness of collective health protection [24, 35].

From the perspective of justificatory attribution, these indicators belong to the collective health goods domain because their primary moral significance lies in evaluating the protection of shared health goods rather than aggregate welfare, fair opportunity, responsibility-sensitive disadvantage, human functioning, or structural disadvantage. Although these indicators may also contribute to other dimensions of health justice, their primary justificatory attribution derives from the distinctive normative importance of collective health protection [10, 24, 25].

The collective health goods domain therefore captures a dimension of health justice that the preceding theories do not treat as their central concern, even though each could in principle offer a supporting justification for the same policies. A health system may maximise overall population health, provide equitable access to healthcare, and appropriately distinguish between brute luck and voluntary choice, yet still fail to protect the collective conditions upon which the health of the entire community depends. For this reason, collective health goods constitute a distinct normative domain within the layered evaluation model, understood as the domain whose primary evaluative object is most directly collective, not as the only theory capable of justifying public health measures [9, 24].

### Functioning (capability justification)

Within the present framework, the relevant normative question is whether a health inequality is unjust because it limits an individual’s capability to achieve valuable functioning. Unlike the preceding domains, which evaluate aggregate welfare, fair opportunity, responsibility, or collective health goods, the capability approach evaluates what individuals are actually able to be and to do [8, 36, 37].

Developed primarily by Sen and further elaborated by Nussbaum, the capability approach argues that justice cannot be adequately assessed through the distribution of resources or healthcare services alone. Individuals differ substantially in their ability to convert similar resources into effective functioning because of differences in health status, disability, age, gender, and social circumstances. Consequently, equal resources do not necessarily produce equal capabilities [36–38].

The primary evaluative object of this domain is human functioning. The corresponding family of health equity indicators therefore includes measures of functional ability, disability, quality of life, independent living, and other indicators that evaluate individuals’ effective capacity to achieve and maintain valuable functionings rather than merely their access to healthcare [8, 38].

From the perspective of justificatory attribution, these indicators belong to the functioning domain because their primary moral significance lies in evaluating effective functioning rather than aggregate welfare, fair opportunity, responsibility-sensitive disadvantage, collective health goods, or structural disadvantage. Their primary justificatory attribution therefore derives from the capability approach’s concern with individuals’ real opportunities to achieve valuable states of being and doing [9, 36, 37].

The functioning domain therefore identifies a distinct dimension of health justice. A health system may provide equitable access to healthcare while substantial differences remain in individuals’ ability to transform healthcare and other resources into effective functioning. For this reason, functioning constitutes a distinct normative domain within the layered evaluation model [8, 9, 38].

### Structural position (structural justice justification)

Within the present framework, the relevant normative question is whether a health inequality is unjust because it arises from enduring structural conditions that systematically disadvantage particular individuals or groups. Whereas the preceding domains primarily evaluate individuals or communities, this domain evaluates the institutional and social structures through which health inequalities are produced and reproduced [9, 23, 39].

Structural theories of justice argue that health inequalities frequently originate from institutional arrangements, social hierarchies, and unequal distributions of power that systematically shape individuals’ opportunities, living conditions, and health trajectories. Justice therefore requires not only improving individual health outcomes but also transforming the structural conditions that generate persistent patterns of disadvantage [9, 23, 39].

The primary evaluative object of this domain is structural disadvantage. Accordingly, the corresponding family of health equity indicators includes measures of socioeconomic inequality, educational attainment, employment conditions, housing quality, neighbourhood deprivation, environmental exposure, discrimination, and other indicators reflecting the social determinants of health [9, 23, 39].

From the perspective of justificatory attribution, these indicators belong to the structural domain because their primary moral significance lies in identifying institutional and social arrangements that systematically generate health disadvantage. Although many of these indicators may also be interpreted within other normative frameworks, their primary justificatory attribution derives from the structural injustice reflected in the social organisation of health [9].

The structural domain completes the layered evaluation model by extending the analysis beyond individual and community characteristics to the institutional environment within which health inequalities emerge and persist. Even when healthcare systems function effectively, opportunities are equitably distributed, responsibility is appropriately recognised, collective health goods are protected, and functioning is improved, persistent structural disadvantage may continue to generate avoidable health inequalities. For this reason, structural position constitutes the final normative domain within the proposed layered evaluation model [9, 23, 39].

## From theory to measurements

The six normative domains reconstructed in the preceding section provide the conceptual foundation for interpreting health equity indicators. The purpose of the proposed framework is not to classify indicators according to their empirical characteristics, but to identify the normative domain that provides the primary moral justification for interpreting the inequalities they reveal. Accordingly, indicator attribution is guided by normative interpretation rather than descriptive similarity [7, 9, 10].

Many health equity indicators may legitimately be interpreted within more than one theory of justice. Mortality, healthcare access, financial hardship, disability, vaccination coverage, and socioeconomic inequalities may each receive different normative interpretations depending on the evaluative object under consideration. Such overlap reflects the multidimensional nature of health justice rather than conceptual inconsistency among indicators [7–9].

The proposed framework therefore attributes indicators according to the normative domain that provides their primary justificatory basis. This principle does not deny alternative interpretations of individual indicators. Rather, it identifies the domain within which an indicator receives its primary normative significance while preserving both conceptual clarity and normative pluralism [9, 10].

The transition from normative theory to empirical measurement consequently requires an explicit interpretive step. Indicators do not possess intrinsic normative meaning independently of the theories through which they are interpreted. They become indicators of health justice only after the evaluative object underlying the observed inequality has been identified [7, 9].

### Indicator attribution principles

The attribution of health equity indicators follows the principle of justificatory attribution, according to which indicators are assigned to the normative domain that provides the primary moral justification for interpreting the inequalities they measure. Attribution therefore depends neither upon historical origin nor empirical application, but upon normative justification [9, 10].

The analytical procedure adopted in this study is termed the Focal Attribution Procedure. Under this procedure, indicator families are attributed to the domain in which their primary evaluative object is most clearly expressed. Although many indicators remain relevant to multiple theories of justice, focal attribution identifies the normative perspective that provides the principal justification for interpreting the measured inequality as unjust [7, 9].

The procedure is intended to clarify rather than eliminate normative plurality. It neither assumes that theories of justice are mutually exclusive nor implies that alternative interpretations are invalid. Instead, it provides an analytical method for organising indicator families according to their primary justificatory basis while preserving the legitimacy of complementary normative interpretations [8, 9].

This approach differs from classifications based solely on empirical characteristics or policy application. Indicators measuring similar empirical phenomena may belong to different normative domains because the inequalities they identify derive their moral significance from different evaluative objects. Conversely, empirically diverse indicators may belong to the same normative domain when they share a common justificatory foundation [9].

The resulting attribution framework is therefore interpretive rather than taxonomic. Its purpose is to establish a systematic relationship between empirical indicators and the normative theories that provide their primary moral justification in the evaluation of health justice [7, 9].

### Indicator domains

The principle of justificatory attribution provides the methodological foundation for assigning health equity indicators to distinct normative domains. Each domain is defined by its primary evaluative object rather than by the empirical characteristics of its indicators. Accordingly, indicators are attributed according to the normative question they address rather than the empirical phenomenon they measure [9].

Although individual indicators may legitimately contribute to more than one conception of justice, the present framework identifies the domain that provides their primary justificatory basis. The resulting classification should therefore be understood as an interpretive framework rather than a definitive taxonomy of health equity indicators [9, 10] (Table 1).

**Table 1:** Normative domains and indicator attribution

Normative Domain	Primary Evaluative Object	Representative Indicator Families	Primary Justificator Basis
Population Outcomes	Aggregate health welfare	Life expectancy, HALE, DALYs, QALYs, burden of disease, preventable mortality, cost-effectiveness	Maximization of aggregation health benefits
Opportunity	Fair opportunity	Universal health coverage, healthcare access, unmet healthcare needs, financial protection, service availability	Protection of fair opportunity
Responsibility	Responsibility-sensitive disadvantage	Behavioural risk, preventable disease, avoidable morbidity, indicators distinguishing brute luck from choice	Compensation for disadvantages arising from brute luck
Collective Health Goods	Shared health protection	Vaccination coverage, communicable disease control, sanitation, environmental health, public health preparedness	Protection of collective health goods
Functioning	Human functioning	Disability, functional status, quality of life, independent living, activities of daily living	Preservation of effective functioning
Structural Position	Structural disadvantage	Education, income, housing, employment, discrimination, neighbourhood conditions, social determinants of health	Elimination of structural injustice

The proposed attribution does not imply that individual indicators cannot be interpreted within alternative normative frameworks. Rather, it identifies the normative domain that provides each indicator’s primary justificatory basis within the present analytical framework. This distinction is essential because many health equity indicators simultaneously reflect multiple dimensions of health justice. Accordingly, focal attribution identifies conceptual priority rather than empirical exclusivity, preserving alternative normative interpretations while clarifying the principal evaluative object that justifies the indicator’s use [7, 9].

#### Population outcomes (utilitarian domain)

Indicators attributed to the population outcomes domain evaluate the extent to which health systems maximise aggregate population health. Their primary evaluative object is aggregate health welfare rather than the distribution of health benefits among individuals. Representative indicators include life expectancy, healthy life expectancy, DALYs, QALYs, burden of disease, preventable mortality, and cost-effectiveness measures. These indicators are attributed to this domain because they primarily evaluate improvements in aggregate health production [19, 21, 29].

Although these indicators may also be interpreted within other normative domains, their primary justificatory attribution derives from the utilitarian objective of maximising aggregate population health. Their principal normative significance therefore lies in evaluating the efficiency with which health resources generate overall health benefit [10, 19, 27].

#### Opportunity (liberal egalitarian domain)

Indicators attributed to the opportunity domain evaluate whether individuals enjoy fair opportunities to obtain the health services necessary to pursue their life plans. Their primary evaluative object is fair opportunity rather than aggregate population health [6, 7, 30].

Representative indicators include universal health coverage, healthcare accessibility, unmet healthcare needs, financial protection, service availability, and equitable utilisation of essential health services. These indicators are attributed to this domain because they primarily identify inequalities that restrict fair opportunity to achieve and maintain health [7, 10, 22].

Although these indicators may also be interpreted within other theories of justice, their primary justificatory attribution derives from the liberal egalitarian commitment to protecting fair opportunity [6, 7].

#### Responsibility (luck egalitarian domain)

Indicators attributed to the responsibility domain evaluate inequalities according to whether they arise from circumstances beyond individual control or from voluntary choice. Their primary evaluative object is responsibility-sensitive disadvantage rather than fair opportunity or aggregate welfare [12, 15–17]. Representative indicators include measures distinguishing unavoidable health disadvantage from inequalities associated with behavioural risk, preventable disease, and other circumstances in which individual responsibility becomes morally relevant. These indicators are attributed to this domain because they primarily evaluate the normative significance of the distinction between brute luck and voluntary choice [12, 32].

Although these indicators may also contribute to other dimensions of justice, their primary justificatory attribution derives from the luck egalitarian principle that responsibility may legitimately influence distributive judgments under appropriate conditions [12, 15].

#### Collective health goods (communitarian domain)

Indicators attributed to the collective health goods domain evaluate health goods that can only be achieved and sustained through collective action. Their primary evaluative object is the protection of collective health goods rather than individual welfare or distributive equality [24, 25, 33]. Representative indicators include vaccination coverage, communicable disease surveillance, environmental sanitation, food safety, emergency preparedness, and public health infrastructure. These indicators are attributed to this domain because they evaluate health conditions whose protection depends upon shared institutions and coordinated social action [24, 35].

Although these indicators may also contribute to other normative domains, their primary justificatory attribution derives from the communitarian commitment to protecting the collective conditions necessary for public health [10, 25].

#### Functioning (capability domain)

Indicators attributed to the functioning domain evaluate individuals’ effective capability to achieve valuable states of being and doing. Their primary evaluative object is human functioning rather than healthcare utilisation or resource distribution [8, 36, 37]. Representative indicators include disability, functional capacity, quality of life, independent living, cognitive functioning, and activities of daily living. These indicators are attributed to this domain because they evaluate individuals’ effective ability to convert available health resources into meaningful functioning [8, 38].

Although these indicators may also be interpreted within alternative normative frameworks, their primary justificatory attribution derives from the capability approach’s concern with effective human functioning as the appropriate object of justice [9, 36, 37].

#### Structural position (structural justice domain)

Indicators attributed to the structural position domain evaluate persistent patterns of disadvantage arising from social, economic, and institutional arrangements. Their primary evaluative object is structural disadvantage rather than individual health status alone [9, 23, 39].Representative indicators include educational attainment, income inequality, employment conditions, housing quality, neighbourhood deprivation, environmental exposure, discrimination, and other measures reflecting the social determinants of health. These indicators are attributed to this domain because they identify institutional and structural conditions that systematically generate health inequalities across populations [9, 39].

Although these indicators may also be interpreted within other normative domains, their primary justificatory attribution derives from structural theories of justice, which locate the principal source of health inequality in enduring patterns of institutional and social disadvantage [9, 23].

### Layered evaluation model

The attribution of health equity indicators to distinct normative domains leads to a Layered Evaluation Model of health justice. Rather than treating health justice as a single measurable construct, the proposed model recognises that different theories of justice identify different evaluative objects and therefore justify different families of health equity indicators. Consequently, no single indicator or family of indicators can adequately capture the full normative content of health justice [7, 9, 10].

The layered evaluation model preserves the conceptual integrity of each normative domain while enabling multiple normative perspectives to inform health policy simultaneously. Population outcomes, fair opportunity, responsibility-sensitive disadvantage, collective health goods, human functioning, and structural disadvantage each represent distinct dimensions of justice that illuminate different aspects of health inequalities. Their combined consideration therefore provides a more comprehensive evaluation of health justice than reliance on any single normative framework [7–9].

Unlike composite indices that aggregate diverse indicators into a single numerical measure, the layered evaluation model preserves the distinction between different evaluative objects. Indicators remain analytically distinct because they address different normative questions. Consequently, apparent disagreement between indicator families should not automatically be interpreted as methodological inconsistency. Rather, it frequently reflects the plurality of legitimate normative perspectives through which health inequalities may be understood [5, 9].

The model therefore provides an interpretive framework for integrating diverse indicator families without reducing their philosophical differences. Instead of asking which indicator provides the correct measure of health justice, the layered evaluation model asks which normative domain provides the most appropriate justification for evaluating the policy question under consideration. This shift from competition among indicators to interpretation through normative domains constitutes the principal methodological contribution of the present framework [7, 9].

The practical application of the layered evaluation model requires a clear distinction between empirical observation and normative evaluation. Empirical indicators identify measurable health inequalities, whereas normative domains explain why those inequalities should be regarded as matters of justice. Health equity assessment therefore proceeds in two complementary stages: empirical identification of health inequalities followed by normative interpretation through the evaluative object that provides their primary justificatory basis. Making this distinction explicit improves both the transparency and the consistency of health equity evaluation [2, 7, 9].

The layered evaluation model is intended neither to replace existing health equity indicators nor to establish a hierarchy among competing theories of justice. Instead, it provides a coherent interpretive framework through which different indicator families can be understood, compared, and justified while preserving normative pluralism. By systematically relating empirical indicators to their primary justificatory foundations, the model offers a more transparent and philosophically coherent approach to the evaluation of health justice [8–10].

#### Relationship to composite and multicriteria approaches

This account of the model’s contribution is not a claim that aggregation itself is illegitimate. It is worth distinguishing several activities that composite indices often blur together: measurement, which records an empirical pattern; monitoring, which tracks that pattern over time; evaluation, which interprets whether a pattern is unjust; aggregation, which combines several evaluations into a summary statistic; and decision-making, which chooses among policies on the basis of that summary. Several established methods carry out aggregation while keeping the value judgements involved explicit rather than implicit. Multicriteria decision analysis (MCDA) structures priority-setting decisions by specifying criteria, weights, and scoring rules openly, so that stakeholders can see and contest the value judgements embedded in a final ranking [40]. Distributional cost-effectiveness analysis (DCEA) extends conventional cost-effectiveness analysis by modelling the distribution of health gains and losses across social groups and applying an explicit, defensible social welfare function to weigh efficiency against equity [41]. Deliberative and procedural approaches, such as Daniels and Sabin’s “accountability for reasonableness,” aggregate competing considerations through a transparent, contestable process rather than a fixed formula [42, 43].

These approaches do not undermine the argument developed here; rather, they illustrate it. Each requires an antecedent judgement about which values are being weighed and why, a judgement that MCDA, DCEA, and deliberative procedures render visible rather than eliminate. The layered evaluation model does not compete with these methods at the level of decision procedure. It operates one step earlier, by clarifying which normative domains are in play before any weighting, scoring, or deliberation begins. A policymaker using MCDA or DCEA must still decide which criteria to include, and the six domains identified here offer one principled basis for that prior decision. The model therefore cannot determine how domains should be traded off against one another in a specific policy decision; it clarifies what is being traded off, and for what normative reasons, leaving the weighting itself to be settled by whichever aggregation method or deliberative process a health system adopts.

## Policy and priority-setting implications

The proposed framework has important implications for health policy and priority-setting because it demonstrates that policy disagreements frequently arise from differences in normative interpretation rather than from disagreements about empirical evidence. Decision-makers often rely on the same empirical data while reaching different policy conclusions because they implicitly adopt different conceptions of justice. Making these normative assumptions explicit improves both the transparency and the consistency of policy deliberation [7, 9, 10].

Conventional approaches to priority-setting frequently assume that health justice can be adequately represented by a single comprehensive indicator or composite index. The present analysis challenges this assumption by demonstrating that different health equity indicators evaluate different normative objects. Consequently, policies that perform well within one normative domain may be evaluated differently within another. Priority-setting should therefore begin by identifying the relevant normative domain before selecting the indicators used to evaluate competing policy options [5, 7, 9].

The proposed framework also offers a systematic approach to interpreting apparent conflicts among health equity indicators. Rather than treating divergent indicators as evidence of methodological weakness, policymakers should first determine whether the indicators evaluate different normative objects. Apparent disagreement between indicators frequently reflects legitimate differences in conceptions of justice rather than deficiencies in empirical measurement [8, 9].

A further implication concerns the interpretation of national and international health equity monitoring systems. Many existing monitoring frameworks combine indicators originating from different normative traditions without explicitly recognising their distinct justificatory foundations. Applying the present framework makes these foundations explicit by identifying the primary justificatory basis of individual indicator families and clarifying the dimensions of justice represented within existing monitoring systems [4, 9, 23].

The proposed framework is not intended to prescribe a single model of priority-setting. Rather, it provides an interpretive framework through which policymakers, researchers, and health institutions can identify the normative assumptions underlying competing approaches to health equity evaluation. By distinguishing empirical measurement from normative interpretation, the framework supports more transparent, consistent, and philosophically grounded policy analysis [7, 9, 10].

### Illustrative application: cost-effectiveness

Cost-effectiveness analysis illustrates the interpretive framework developed in this article by demonstrating how the same policy decision may receive different normative evaluations depending on the evaluative object under consideration. Although cost-effectiveness measures are frequently presented as objective tools for priority-setting, their normative significance depends upon the conception of justice through which they are interpreted [7, 19, 21].

Within the utilitarian domain, cost-effectiveness analysis serves as a principal instrument for maximising aggregate population health. Interventions that generate the greatest health gain relative to available resources receive priority because they maximise overall health benefit. Indicators such as cost per QALY or cost per DALY therefore derive their primary justificatory basis from the utilitarian objective of maximising aggregate health welfare [19, 21, 27].

Within the opportunity domain, the same policy is evaluated according to whether it preserves fair opportunity to obtain essential healthcare. An intervention that is highly cost-effective may nevertheless be regarded as unjust if it systematically restricts access to services necessary for individuals to maintain normal functioning and participate fully in society. Consequently, protecting fair opportunity may justify priority-setting decisions that differ from those supported by utilitarian evaluation alone [6, 7].

Within the responsibility domain, priority-setting depends upon whether health disadvantage results from brute luck or voluntary choice. Cost-effectiveness analysis cannot by itself determine the legitimacy of this distinction because the relevance of individual responsibility is a normative rather than an empirical question. Responsibility-sensitive evaluation may therefore support different allocation decisions even when the underlying evidence concerning costs and effectiveness remains unchanged [12, 15, 16].

Within the collective health goods domain, cost-effectiveness is evaluated according to its contribution to protecting health goods that can only be achieved through collective action. Vaccination programmes, communicable disease control, environmental health, and emergency preparedness generate benefits extending beyond individual recipients. Their justification therefore depends not only on aggregate efficiency but also on the protection of shared conditions necessary for public health [10, 24, 25].

Within the functioning domain, healthcare interventions are evaluated according to their capacity to preserve or restore effective human functioning. An intervention may therefore deserve priority because it substantially improves an individual’s ability to achieve valuable functionings, even when its contribution to aggregate population health is comparatively limited. Functional achievement rather than aggregate efficiency becomes the primary evaluative object [36–38].

Within the structural domain, priority-setting focuses on reducing persistent structural disadvantage. Policies addressing socioeconomic inequality, environmental deprivation, discrimination, or other structural determinants of health may therefore receive priority even when they produce smaller short-term improvements in aggregate health outcomes. Their primary justification lies in transforming the structural conditions that systematically generate health inequalities [9, 23, 39].

This example illustrates that disagreements concerning cost-effectiveness frequently arise from differences in normative interpretation rather than disagreements concerning empirical evidence. Cost-effectiveness analysis therefore represents one important dimension of health justice rather than a comprehensive criterion for distributive decision-making. Identifying the relevant normative domain before interpreting cost-effectiveness evidence enables more transparent, consistent, and philosophically coherent priority-setting decisions [7, 9, 10].

## Objections and replies

A potential objection to the proposed framework is that many health equity indicators can legitimately be interpreted within more than one theory of justice. Mortality, disability, healthcare access, financial hardship, and socioeconomic deprivation may each receive utilitarian, egalitarian, capability-based, communitarian, or structural interpretations. Consequently, attributing indicators to distinct normative domains may appear arbitrary [7–9].

The present framework does not deny the legitimacy of multiple interpretations. Rather, it distinguishes between alternative normative interpretations and primary justificatory attribution. Indicators are not attributed because alternative interpretations are excluded, but because one normative domain provides the principal moral justification for interpreting the inequality they reveal as unjust. Focal attribution therefore establishes conceptual priority rather than interpretive exclusivity [9, 10].

A second objection concerns the analytical separation of liberal egalitarianism and luck egalitarianism. Since both belong to the broader egalitarian tradition, distinguishing them as separate domains may appear unnecessary. However, the distinction reflects differences in their primary evaluative objects rather than their historical origins. Liberal egalitarianism evaluates inequalities primarily in terms of fair opportunity, whereas luck egalitarianism evaluates them according to the distinction between brute luck and voluntary choice. Because these theories provide different justificatory foundations for interpreting health inequalities, they also justify different families of health equity indicators. Their separation therefore serves analytical clarification rather than taxonomic revision [6, 7, 12, 15–17].

A further objection concerns the inclusion of communitarian justice as an independent normative domain, given that public health interventions can also be justified within utilitarian or rights-based frameworks. As explained in Section "Collective health goods (communitarian justification)", the framework does not dispute this: it retains the domain because the collective, non-individually-securable character of these goods is not the primary concern of the other theories, not because they are incapable of supporting the same policies [10, 24, 25].

Finally, it may be argued that the proposed framework merely reorganises existing indicators without generating new empirical knowledge. This criticism, however, misunderstands the objective of the study. The contribution of the framework is methodological and interpretive rather than empirical. It does not introduce new indicators; instead, it provides a systematic method for explaining why existing indicators receive different normative interpretations and why they frequently support different policy recommendations [7, 9].

## Justification of indicator attribution

The principle of justificatory attribution developed in this article rests upon the distinction between empirical observation and normative evaluation. Health equity indicators identify measurable differences in health status, healthcare, or the social conditions influencing health. They do not, however, determine why those differences should be regarded as unjust. That determination depends upon the normative framework through which empirical inequalities are interpreted [7, 9].

Accordingly, indicator attribution is grounded in the primary evaluative object identified by each normative domain. Population outcome indicators derive their primary normative significance from aggregate health welfare; opportunity indicators from fair opportunity; responsibility indicators from responsibility-sensitive disadvantage; collective health indicators from the protection of collective health goods; functioning indicators from effective human functioning; and structural indicators from structural disadvantage. The proposed attribution therefore links each family of indicators to the normative object that provides its primary moral justification [7–9].

The proposed attribution represents an interpretive rather than an empirical classification. Its purpose is not to assign indicators exclusively to a single theory of justice, but to identify the normative framework that provides the strongest justification for interpreting the inequalities they reveal. Alternative interpretations remain possible because many indicators illuminate more than one dimension of health justice. The framework therefore preserves normative pluralism while establishing a coherent basis for comparative analysis [9, 10].

Organising indicators according to their primary justificatory basis also clarifies the relationships among competing theories of justice. Similarities and differences between normative frameworks become more transparent because they are analysed through their evaluative objects rather than through their empirical indicators alone. Apparent conflicts among indicator families can therefore be understood as reflecting differences in normative interpretation rather than inconsistencies in empirical measurement [5, 9].

The principle of justificatory attribution thus provides the methodological bridge between normative theory and empirical measurement. It explains how health equity indicators acquire normative meaning and establishes a systematic interpretive framework through which different indicator families can be compared without reducing the plurality of conceptions of health justice [7, 9, 10].

## Conclusion

This article has argued that health equity cannot be fully understood or evaluated through a single family of indicators alone, because different theories of justice identify different evaluative objects. This does not imply that measurement, monitoring, or aggregation are illegitimate; transparent multicriteria and distributional methods can incorporate several of these domains simultaneously. It implies that any such aggregation involves antecedent normative choices about which domains to include and how to weigh them, choices this article has sought to make explicit rather than assume. Persistent disagreement over health equity measurement therefore reflects normative pluralism rather than deficiencies in empirical methodology. Health equity indicators do not merely provide alternative measures of the same concept of justice; they evaluate different normative dimensions of health inequality [7, 9, 10].

To address this problem, the study developed a comparative interpretive framework that reconstructs six normative domains of health justice and attributes health equity indicators according to their primary justificatory basis. By introducing the principles of justificatory attribution and the Focal Attribution Procedure, the proposed framework establishes a systematic method for relating empirical indicators to the normative theories that explain their moral significance. The resulting Layered Evaluation Model preserves the conceptual distinction between different dimensions of justice while allowing them to contribute simultaneously to health policy evaluation [7–9].

The proposed framework neither replaces existing health equity indicators nor establishes a hierarchy among competing theories of justice. Instead, it demonstrates that different indicator families address different normative questions and should therefore be interpreted within the theoretical frameworks that provide their primary justification. Making these normative foundations explicit improves the conceptual transparency of health equity assessment and provides a more coherent basis for comparing competing policy recommendations [9, 10].

The principal contribution of this study is therefore methodological rather than empirical. Rather than treating disagreement among health equity indicators as a problem to be eliminated, the framework interprets such disagreement as the inevitable consequence of competing conceptions of justice and provides a systematic method for understanding that plurality. By explicitly linking empirical indicators to their underlying normative justifications, the proposed framework offers a more conceptually coherent, philosophically grounded, and analytically transparent approach to the interpretation and evaluation of health justice [7–9].

## Data Availability

No datasets were generated or analysed during the current study.
